# Supplementary material for: Cumulative effect of PM2.5 components is larger than the effect of PM2.5 mass on child health in India
Source: Nat Commun. 2023 Oct 31;14:6955. doi: 10.1038/s41467-023-42709-1 (PMC10618175; doi:10.1038/s41467-023-42709-1)
Supplement: Supplementary file 1 — Supplementary Information file [file 41467_2023_42709_MOESM1_ESM.pdf]

Cumulative effect of $PM_{2.5}$ components is larger than the effect of $PM_{2.5}$ mass on child health in India

†Ekta Chaudhary[1], †Franciosalgeo George[2], Aswathi Saji[2], *Sagnik Dey[1,3,4], *Santu Ghosh[5], Tinku Thomas[5], Anura V Kurpad[6], Sumit Sharma[7], Nimish Singh[1,7], Shivang Agarwal[7,8], Unnati Mehta[9]

[1] Centre for Atmospheric Sciences, Indian Institute of Technology Delhi, New Delhi, India
[2] Division of Epidemiology, Biostatistics, and Population Health, St John's Research Institute, Bangalore, India
[3] Centre of Excellence for Research on Clean Air, IIT Delhi, New Delhi, India
[4] School of Public Policy, IIT Delhi, New Delhi, India
[5] Department of Biostatistics, St John's Medical College, Bengaluru, India
[6] Department of Physiology, St John's Medical College, Bengaluru, India
[7] TERI, New Delhi, India
[8] Johns Hopkins University, Maryland, U.S.A
[9] Harvard T.H. Chan School of Public Health, Boston, U.S.A

†Equal contribution
*Corresponding Authors:
Sagnik Dey, Centre for Atmospheric Sciences, Indian Institute of Technology Delhi, New Delhi, India.
E-mail: sagnik@cas.iitd.ac.in
Contact: 011-2659-1315
Santu Ghosh, Department of Biostatistics, St John's Medical College, Bengaluru, India.
E-mail: santu.g@stjohns.in
Contact: +91-8049466123

Contents:

Supplementary Figures 1-5

Supplementary Table 1-3

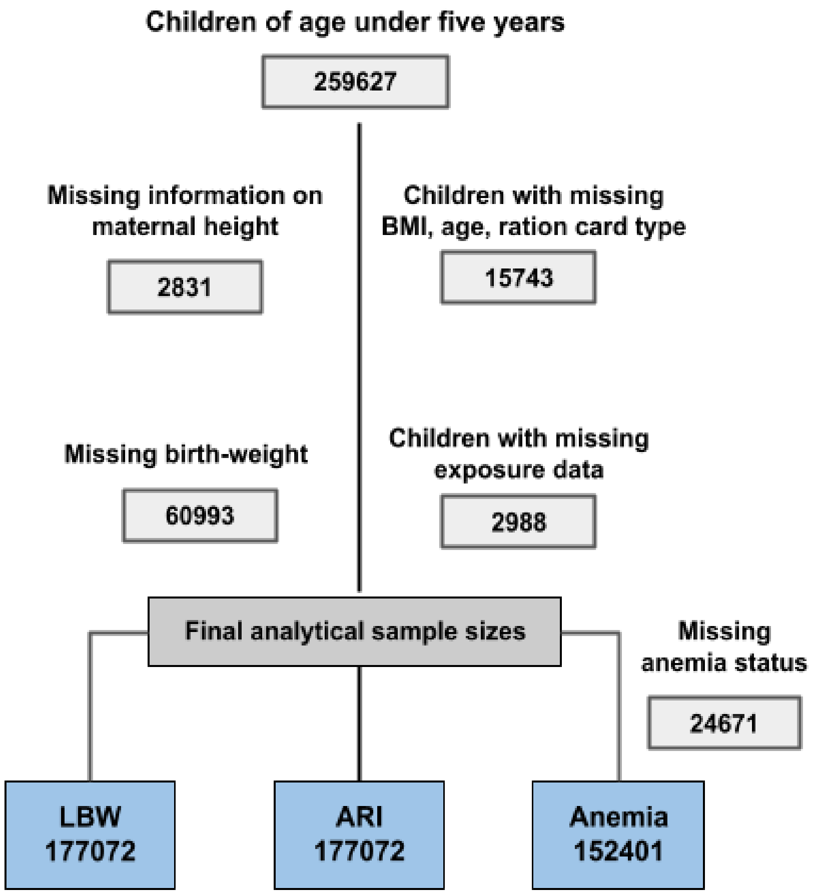

Supplementary Figure 1: Schematic consort diagram demonstrating the exclusion criteria of observations in the NFHS-4 dataset.

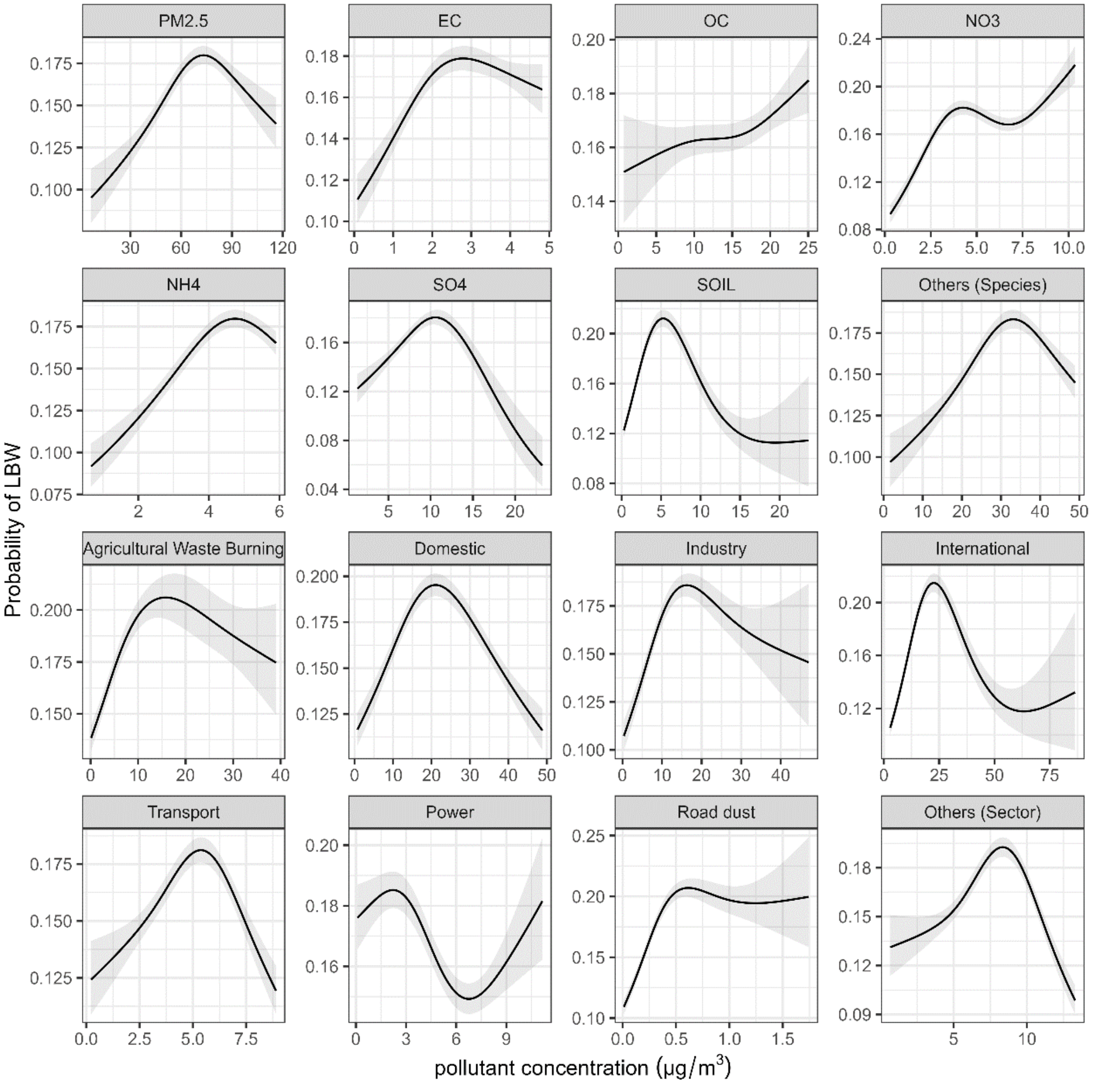

Supplementary Figure 2: Exposure-response curves for LBW against components and sectors contributing to $PM_{2.5}$. The shaded region represents 95% confidence interval. Source data are provided as a Source Data file.

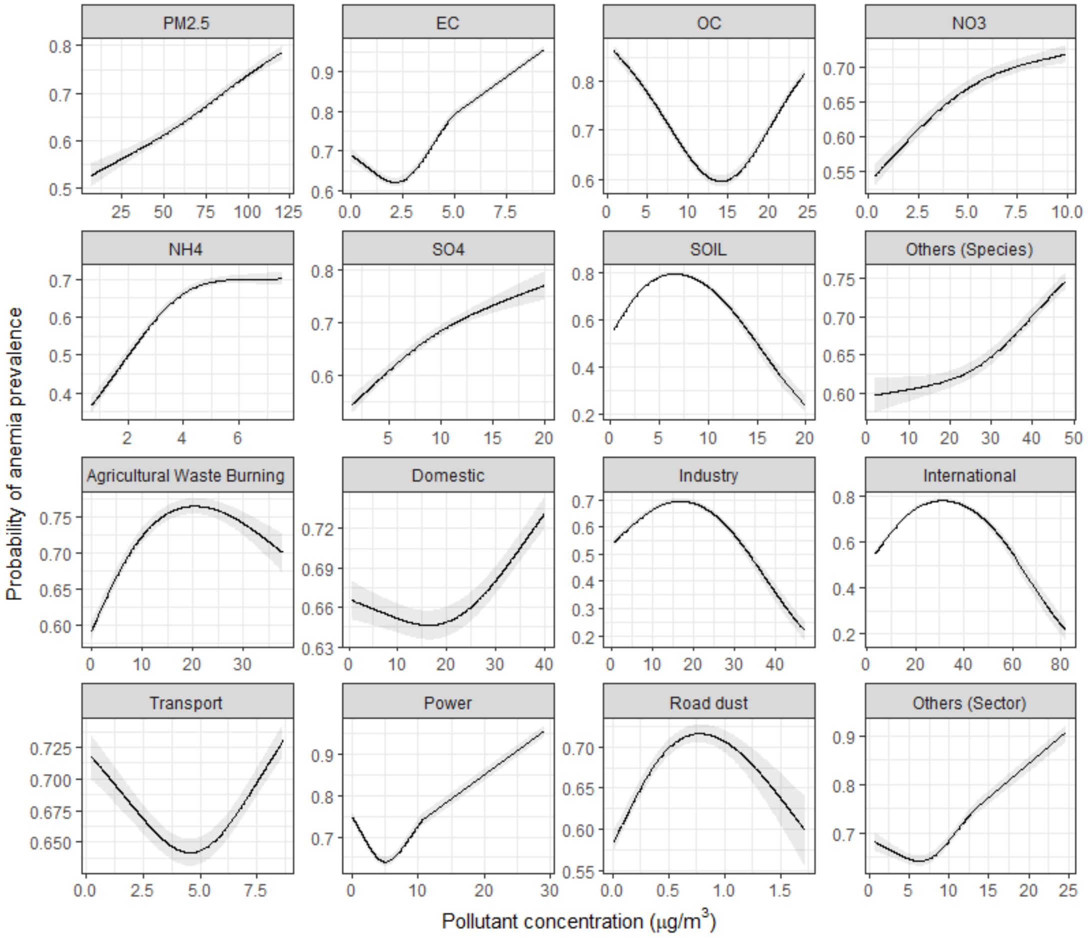

Supplementary Figure 3: Exposure-response curves for anaemia against components and sectors contributing to $PM_{2.5}$. The shaded region represents 95% confidence interval. Source data are provided as a Source Data file.

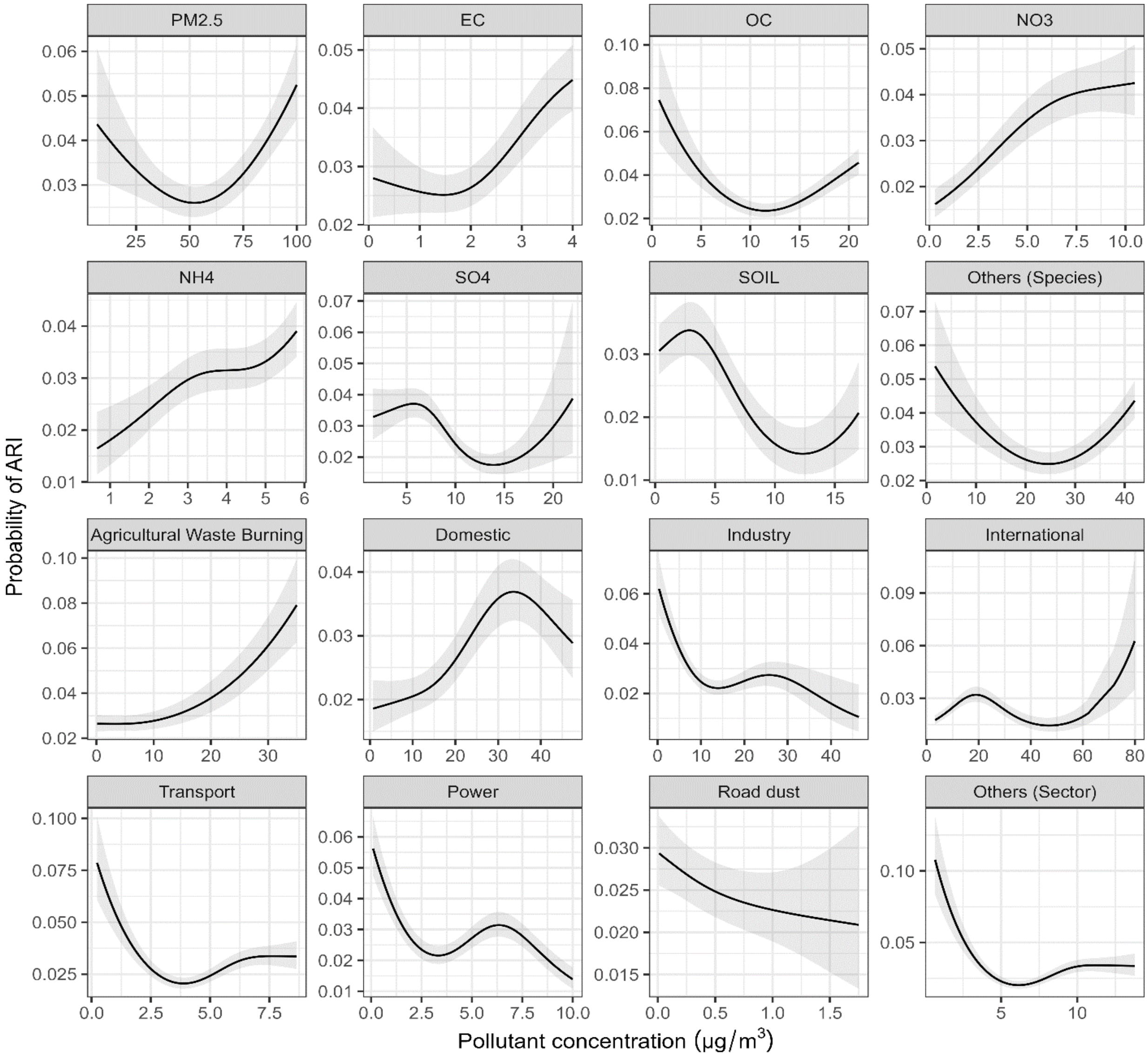

Supplementary Figure 4: Exposure-response curves for ARI against components and sectors contributing to $PM_{2.5}$. The shaded region represents 95% confidence interval. Source data are provided as a Source Data file.

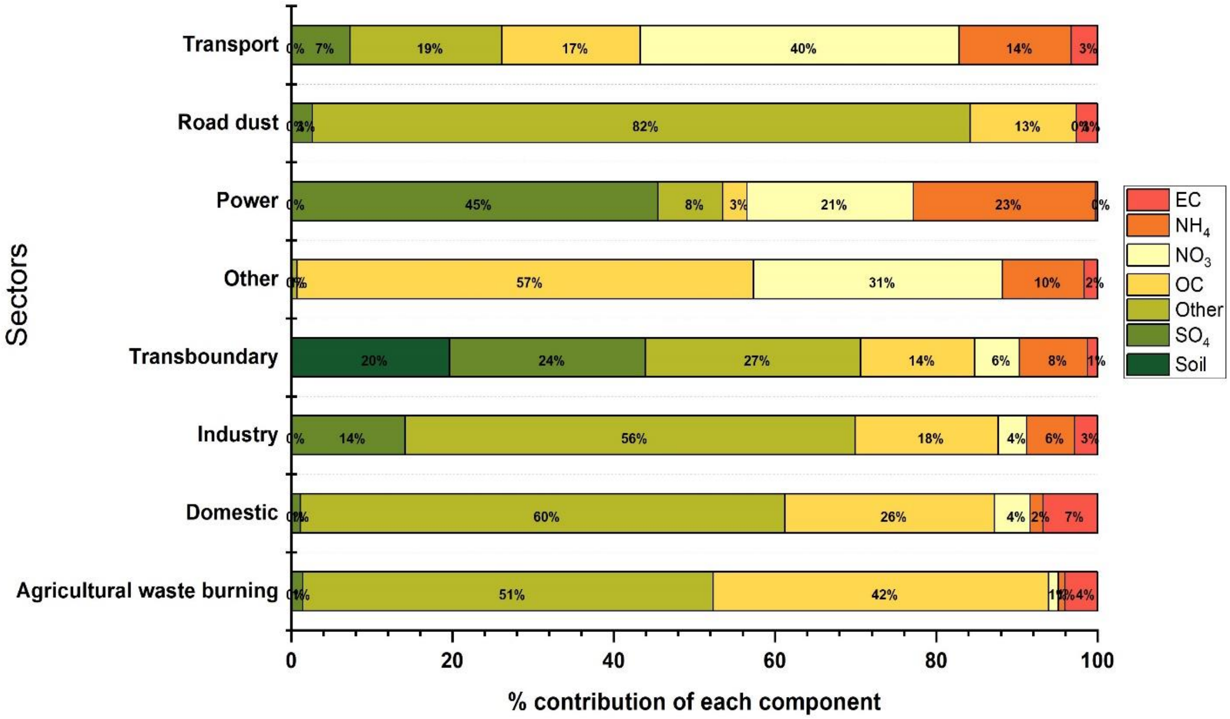

Supplementary Figure 5: Overall percent contribution of each component in each sector. Sector 'other' involves refused burning, construction, crematoria, NH_3, biogenic emissions, and evaporative non-methane volatile organic compounds (NMVOCs). Whereas component 'other' involves chloride, sodium, magnesium, calcium, soil, water molecules, and unspecified species. Source data are provided as a Source Data file.

Supplementary Table 1- Prevalence of LBW, ARI and anaemia of U5 children in India stratified by potential predictors.

Variable	Prevalence (95% CI) in %		
	**ARI**	**LBW**	**Anaemia**
**Overall**	2.8 (2.72, 2.88)	16.6 (16.43, 16.77)	56.8 (56.64, 57.14)
**Sex of Child**			
Male (n= 92848)	2.9 (2.7, 3.01)	15.5 (15.27, 15.73)	56.9 (56.56, 57.24)
Female (n= 84224)	2.5 (2.39, 2.61)	17.8 (17.54, 18.06)	56.5 (56.44, 57.16)
**Mother's Education**			
No education (n= 41627)	2.6 (2.45, 2.75)	18.6 (18.23, 18.97)	64.8 (64.31, 65.29)
Primary (n= 24469)	3.1 (2.88, 3.32)	18.0 (17.52, 18.48)	58.9 (58.24, 59.56)
Secondary (n= 90267)	2.8 (2.69, 2.91)	16.0 (15.76, 16.24)	54.1 (53.75, 54.45)
Higher (n= 20709)	2.6 (2.38, 2.82)	13.2 (12.74, 13.66)	50.2 (49.46, 50.94)
**Wealth index**			
Poorest (n= 35662)	2.9 (2.73, 3.07)	18.3 (17.90, 18.70)	63.6 (63.06, 64.14)
Poor (n= 39257)	2.9 (2.73, 3.07)	17.1 (16.73, 17.47)	58.0 (57.47, 58.53)
Middle (n= 38133)	2.8 (2.63, 2.97)	16.4 (16.03, 16.77)	56.0 (55.46, 56.54)
Rich (n= 34345)	2.5 (2.33, 2.67)	16.1 (15.71, 16.49)	53.3 (52.73, 53.87)
Richest (n= 29675)	2.6 (2.42, 2.78)	14.4 (14.00, 14.80)	52.5 (51.89, 53.11)
**Religion**			
Hindu (n= 133085)	2.6 (2.51, 2.69)	17.4(17.2, 17.6)	56.7 (55.50, 57.90)
Muslim (n= 24214)	3.7 (3.46, 3.94)	16.5 (16.03, 16.97)	58.5 (58.22, 58.78)
Christian (n= 12177)	2.5 (2.22, 2.78)	8.9 (8.39, 9.41)	58.7 (58.03, 59.37)
Others (n= 7596)	3.1 (2.71, 3.49)	14.5 (13.71, 15.29)	35.8 (34.87, 36.73)
**Household has BPL card**			
BPL (n= 67048)	2.5 (2.38, 2.62)	16.7 (16.42, 16.98)	59.2 (58.80, 59.60)
Non-BPL (n= 110024)	2.9 (2.80, 3.00)	16.5 (16.28, 16.72)	55.5 (55.18, 55.82)
Place of residence			
Rural (n= 129357)	2.9 (2.81, 2.99)	16.7 (16.5, 16.9)	54.1 (53.62, 54.58)
Urban (n= 47715)	2.4 (2.26, 2.54)	16.2 (15.87, 16.53)	57.9 (57.61, 58.19)
**Cooking Fuel**			
Clean fuel (n= 61601)	2.5 (2.38, 2.62)	15.1 (14.82, 15.38)	53.0 (52.58, 53.42)
Solid fuel (n= 114064)	2.9 (2.80, 3.00)	17.3 (17.08, 17.52)	59.0 (58.69, 59.31)
Kerosine (n= 1407)	3.3 (2.37, 4.23)	17.8 (15.80, 19.80)	55.3 (52.48, 58.12)
**Smoking Behaviour**			
No (n= 90587)	2.6 (2.5, 2.7)	16.2 (15.96, 16.44)	56.7 (56.35, 57.05)
Yes (n= 86485)	2.9 (2.79, 3.01)	16.9 (16.65, 17.15)	57.1 (56.74, 57.46)
**Gravida**			
Primi (n= 74222)	2.9 (2.78, 3.02)	15.6 (15.34, 15.86)	54.1 (53.72, 54.48)

Multi (n= 102850)	2.7 (2.6, 2.8)	17.2 (16.97, 17.43)	58.9 (58.57, 59.23)
**PM2.5**			
<Q1 (n= 44268)	2.4 (2.26, 2.54)	13.6 (13.28, 13.92)	46.1 (45.60, 46.60)
Q1 - Q2 (n= 44268)	2.2 (2.06, 2.34)	17.1 (16.75, 17.45)	58.4 (57.91, 58.89)
Q2 - Q3 (n= 44268)	2.8 (2.65, 2.95)	18.3 (17.94, 18.66)	59.1 (58.61, 59.59)
>Q3 (n= 44268)	3.6 (3.43, 3.77)	17.3 (16.95, 17.65)	64.0 (63.52, 64.48)
**Mother's age in year**			
<20 (n= 4763)		19.9 (18.77, 21.03)	64.9 (63.22, 66.58)
20-35 (n= 157778)		16.6 (16.42, 16.78)	57.3 (57.04, 57.56)
>35 (n= 14531)		15 (14.42, 15.58)	50.8 (49.95, 51.65)
**BMI of mother**			
Under weight (n= 40500)		20.2 (19.81, 20.59)	62.3 (61.8, 62.8)
Normal weight (n= 108385)		15.9 (15.68, 16.12)	56.5 (56.18, 56.82)
Overweight (n= 21692)		14.1 (13.64, 14.56)	50.2 (49.48, 50.92)
Obesity (n= 6495)		13.7 (12.86, 14.54)	50.9 (49.6, 52.2)
**Per capita Dietary Iron intake of the household(mg)**			
0-15 (n= 109172)			52.7 (52.37, 53.03)
15-30 (n= 65594)			63 (62.61, 63.39)
30-50 (n= 2306)			62.4 (59.82, 64.98)
**Mother anaemia**			
yes (n= 99442)			63.9 (63.58, 64.22)
no (n= 77630)			53.5 (53.17, 53.83)

Supplementary Table 2: Distribution of $PM_{2.5}$ exposure along with its components and sectoral contribution at PSU level (total PSU numbers are 27828). All units are in μg/m³.

Pollutant	Mean	Median	SD	Q1	Q3
$PM_{2.5}$	63.41	61.78	15.87	51.72	78.45
EC	2.29	1.98	0.92	1.56	3.14
OC	14.09	13.6	3.97	10.81	17.92
NO_3	4.49	3.74	2.29	2.59	6.71
NH_4	3.98	3.97	0.97	3.31	4.71
SO_4	7.73	7.99	2.08	6.15	9.05
SOIL	2.50	2.03	2.00	1.20	3.10
Others	28.71	27.77	7.90	23.00	35.96
Agriculture	5.10	4.09	4.44	2.51	6.04
Domestic	17.7	14.17	8.92	11.11	22.47
Industry	10.45	9.52	4.79	7.72	12.37
International	13.9	12.01	6.73	9.77	16.12
Others (sector)	7.21	6.84	2.02	5.74	8.86
Power	4.00	3.94	1.88	2.52	5.00
Road dust	0.34	0.30	0.20	0.21	0.40
Transport	4.72	4.57	1.41	3.69	5.91

Supplementary Table 3: WRF-CMAQ Model set-up details

**Data/scheme type**	**Options**
**Model resolution**	36 x 36 km with 25 vertical levels
**Meteorology data used**	ECMWF's ERA-5
**Simulation period**	The model was simulated from 1st Jan 2016 to 31st Dec 2016.
**Micro Physics Options in WRF**	WSM three-class simple ice scheme[1]
**Shortwave radiation in WRF**	Dudhia Shortwave Scheme[2]
**Longwave radiation in WRF**	RRTM Longwave Scheme[3]
**Surface layer**	Revised MM5 Monin-Obukhov scheme[4]
**Land Surface Options in WRF**	Unified Noah Land Surface Model scheme[5]
**Boundary layer Options in WRF**	ACM2[6]
**Cumulus Options in WRF**	Kain-Fritsch (new Eta) scheme[7]
**Chemical mechanism used in CMAQ**	CB6r3_ae7_aq (CB6r3 - Carbon Bond 6 version r3; ae7_aq module - CMAQ's aero7 for treatment of SOA set up for standard cloud chemistry)[8,9]
**Emission inventory resolution**	36x36 km
**Emissions of neighbouring countries within the study domain**	ECLIPSE (version 5) database of IIASA
**Transboundary pollutant outside the study domain**	Community Atmosphere Model with Chemistry (CAM-chem) model

Supplementary References

1. Hong, S.-Y., Dudhia, J. & Chen, S.-H. A Revised Approach to Ice Microphysical Processes for the Bulk Parameterization of Clouds and Precipitation. *Monthly Weather Review* **132**, 103–120 (2004).

2. Dudhia, J. Numerical Study of Convection Observed during the Winter Monsoon Experiment Using a Mesoscale Two-Dimensional Model. *Journal of the Atmospheric Sciences* **46**, 3077–3107 (1989).
3. Mlawer, E. J., Taubman, S. J., Brown, P. D., Iacono, M. J. & Clough, S. A. Radiative transfer for inhomogeneous atmospheres: RRTM, a validated correlated-k model for the longwave. *Journal of Geophysical Research: Atmospheres* **102**, 16663–16682 (1997).
4. Jiménez, P. A. *et al.* A Revised Scheme for the WRF Surface Layer Formulation. *Monthly Weather Review* **140**, 898–918 (2012).
5. Chen, F. & Dudhia, J. Coupling an Advanced Land Surface–Hydrology Model with the Penn State–NCAR MM5 Modeling System. Part I: Model Implementation and Sensitivity. *Monthly Weather Review* **129**, 569–585 (2001).
6. Pleim, J. E. A Combined Local and Nonlocal Closure Model for the Atmospheric Boundary Layer. Part I: Model Description and Testing. *Journal of Applied Meteorology and Climatology* **46**, 1383–1395 (2007).
7. Kain, J. S. The Kain–Fritsch Convective Parameterization: An Update. *Journal of Applied Meteorology and Climatology* **43**, 170–181 (2004).
8. Yarwood, G. *et al. Updates to the Carbon Bond Mechanism for Version 6 (CB6), in: 9th Annual CMAS Conference, Chapel Hill, NC, 11–13 October 2010*. 1–4 (2010).
9. Emery, C., Jung, J., Koo, B. & Yarwood, G. *Improvements to CAMx Snow Cover Treatments and Carbon Bond Chemical Mechanism for Winter Ozone, Tech. rep., Ramboll Environ*. (2015).
