# Supplementary material for: Cumulative effect of PM2.5 components is larger than the effect of PM2.5 mass on child health in India
Source: Nat Commun. 2023 Oct 31;14:6955. doi: 10.1038/s41467-023-42709-1 (PMC10618175; doi:10.1038/s41467-023-42709-1)
Supplement: Supplementary file 3 — Reporting Summary [file 41467_2023_42709_MOESM3_ESM.pdf]

nature portfolio

Corresponding author(s): Sagnik Dey; Santu Ghosh

Last updated by author(s): Jul 4, 2023

Reporting Summary

Nature Portfolio wishes to improve the reproducibility of the work that we publish. This form provides structure for consistency and transparency in reporting. For further information on Nature Portfolio policies, see our Editorial Policies and the Editorial Policy Checklist.

Statistics

For all statistical analyses, confirm that the following items are present in the figure legend, table legend, main text, or Methods section.

n/a	Confirmed	
x		The exact sample size (n) for each experimental group/condition, given as a discrete number and unit of measurement
x		A statement on whether measurements were taken from distinct samples or whether the same sample was measured repeatedly
x		The statistical test(s) used AND whether they are one- or two-sided<br>*Only common tests should be described solely by name; describe more complex techniques in the Methods section.*
	x	A description of all covariates tested
	x	A description of any assumptions or corrections, such as tests of normality and adjustment for multiple comparisons
	x	A full description of the statistical parameters including central tendency (e.g. means) or other basic estimates (e.g. regression coefficient) AND variation (e.g. standard deviation) or associated estimates of uncertainty (e.g. confidence intervals)
x		For null hypothesis testing, the test statistic (e.g. F, t, r) with confidence intervals, effect sizes, degrees of freedom and P value noted<br>*Give P values as exact values whenever suitable.*
x		For Bayesian analysis, information on the choice of priors and Markov chain Monte Carlo settings
x		For hierarchical and complex designs, identification of the appropriate level for tests and full reporting of outcomes
x		Estimates of effect sizes (e.g. Cohen's d, Pearson's r), indicating how they were calculated

Our web collection on statistics for biologists contains articles on many of the points above.

Software and code

Policy information about availability of computer code

Data collection	All the collected datasets including survey data (NFHS-4) can be directly accessed from the accession codes given in the data availability statement
Data analysis	All data analyses were done using R software (version 4.1.2) -a publicly available statistical software (https://www.r-project.org/). The R codes are available at https://figshare.com/s/830691d49ae8fe7c4b6b

For manuscripts utilizing custom algorithms or software that are central to the research but not yet described in published literature, software must be made available to editors and reviewers. We strongly encourage code deposition in a community repository (e.g. GitHub). See the Nature Portfolio guidelines for submitting code & software for further information.

Data

Policy information about availability of data

All manuscripts must include a data availability statement. This statement should provide the following information, where applicable:

- Accession codes, unique identifiers, or web links for publicly available datasets
- A description of any restrictions on data availability
- For clinical datasets or third party data, please ensure that the statement adheres to our policy

The survey data that supports the finding of this study is available in a public repository- https://www.dhsprogram.com/data/available-datasets.cfm. Processed exposure and health data along with code used in the analysis are available at https://figshare.com/s/830691d49ae8fe7c4b6b. The model dataset (original) used in this study is available from the corresponding author upon reasonable request.

Research involving human participants, their data, or biological material

Policy information about studies with human participants or human data. See also policy information about sex, gender (identity/presentation), and sexual orientation and race, ethnicity and racism.

Reporting on sex and gender	The survey data (quantitative) used in this study includes background information of children under age five years across India. Authors have not collected this data directly from the participants. Its a secondary data procured from DHS website. This survey data provides information on biomarkers, anthropometric measurements, and household characteristics. We have considered overall children population that includes both male and female sexes.
Reporting on race, ethnicity, or other socially relevant groupings	Our study does not include race, ethnicity, or socially relevant groupings
Population characteristics	Our study involves following individual level characteristics of children aged under five years- sex of child, anthropometry measurements, maternal characteristics, and household related information such as socio-economic status, second hand smoke, cooking fuel type.
Recruitment	We used secondary health dataset (NFHS-4) which was generated using questionnaire based survey. NFHS-4 is a stratified sample selected in two stages from the sampling frame. Stratification was done by segregating each district into urban and rural areas. In the first stage of sample selection, 28,586 primary sampling units (PSUs) were selected. Random selection of households for interview was performed in these PSUs. Our initial sample size of children from NFHS-4 was 259627 children across 640 districts covering 29 states and 6 union territories of India. 259627 observations were then processed and the final analytical sample size came down to 177072 for ARI and LBW, whereas for anaemia it was 152401.
Ethics oversight	NFHS-4 is a nationally representative survey for India available at DHS website. Multiple organizations and institutes were involved in this survey program. International Institute for Population Sciences (IIPS), Mumbai, is the nodal agency for NFHS surveys.

Note that full information on the approval of the study protocol must also be provided in the manuscript.

Field-specific reporting

Please select the one below that is the best fit for your research. If you are not sure, read the appropriate sections before making your selection.

☐ Life sciences ☒ Behavioural & social sciences ☐ Ecological, evolutionary & environmental sciences

For a reference copy of the document with all sections, see nature.com/documents/nr-reporting-summary-flat.pdf

Behavioural & social sciences study design

All studies must disclose on these points even when the disclosure is negative.

Study description	We examined the differential impacts of exposure to ambient PM2.5 and its components on children population in India through a cross-sectional study. We showed that every 10 ug m-3 increase in PM2.5 exposure, anaemia, acute respiratory infection, and low birth weight prevalence increased by 10% (95% uncertainty interval, UI: 9-11), 11% (8-13), and 5% (4-6), respectively, among children in India. We found that the total PM2.5 mass as a surrogate marker for air pollution exposure could substantially underestimate the true composite impact of different components of PM2.5. We further assess the association with species and sectoral PM2.5 to characterize the relative importance of sectoral interventions to provide a better policy guidance. Our findings provide key indigenous evidence to prioritize control strategies for reducing exposure to more toxic species for greater child health benefits in India.
Research sample	We retrieved NFHS-4 data that gives information on household and individual sociodemographic characteristics, anthropometric and blood biochemistry from 259627 children across 640 districts covering 29 states and 6 union territories of India. The sample covers children population, both male and female, that is chosen on the basis of the highest anemia, low birth weight, and acute respiratory infection burden globally.

Sampling strategy	NFHS-4 is a stratified two-stage sample data. The sampling frame for the selection of Primary Sampling Units (PSU) was the 2011 census. PSUs in rural areas were villages, and Census Enumeration Blocks (CEBs) were in urban areas. PSUs with less than 40 households were combined with the closest PSU. Probability Proportional to Size (PPS) sampling was used to select the final PSUs. Selected PSUs with more than 300 households were divided into segments of 100-150 households, and two segments were selected at random with probability proportional to segment size. In the second stage, 22 households were selected from each rural and urban cluster using systematic sampling. Data collection was carried out using various questionnaires. Our study utilizes children population with 259627 observations. out of these 259627 observations, 82555 observations had missing information.
Data collection	We rely on survey questionnaire data collected based on sampling protocol mentioned above. The NFHS-4 survey gives information on household and individual sociodemographic characteristics, anthropometric and blood biochemistry from 259627 children across 640 districts covering 29 states and 6 union territories of India. All these informations were collected by skilled interviewers using standard questionnaires.
Timing	The study was based on health survey data NFHS-4 collected between 20th January 2015 and 4th December 2016
Data exclusions	We made some exclusions on the sample size based on missing information on maternal characteristics, children BMI, age, birth-weight, exposure data and other covariates. There are evidences from literature (mentioned in the manuscript) that support such exclusions.
Non-participation	82555 observations/participants had missing exposure and other risk factors data, therefore the final observation used for LBW and ARI was 177072 and for anaemia it was 152401 (after excluding missing hemoglobin measurements) .
Randomization	The study is not a clinical trial, rather a retrospective cohort study based on cross sectional national survey data. Sample in the survey was selected by multi stage cluster sampling. Therefore this section is not relevant for this study

Reporting for specific materials, systems and methods

We require information from authors about some types of materials, experimental systems and methods used in many studies. Here, indicate whether each material, system or method listed is relevant to your study. If you are not sure if a list item applies to your research, read the appropriate section before selecting a response.

Materials & experimental systems

n/a	Involved in the study
☒	☐ Antibodies
☒	☐ Eukaryotic cell lines
☒	☐ Palaeontology and archaeology
☒	☐ Animals and other organisms
☒	☐ Clinical data
☒	☐ Dual use research of concern
☒	☐ Plants

Methods

n/a	Involved in the study
☒	☐ ChIP-seq
☒	☐ Flow cytometry
☒	☐ MRI-based neuroimaging
